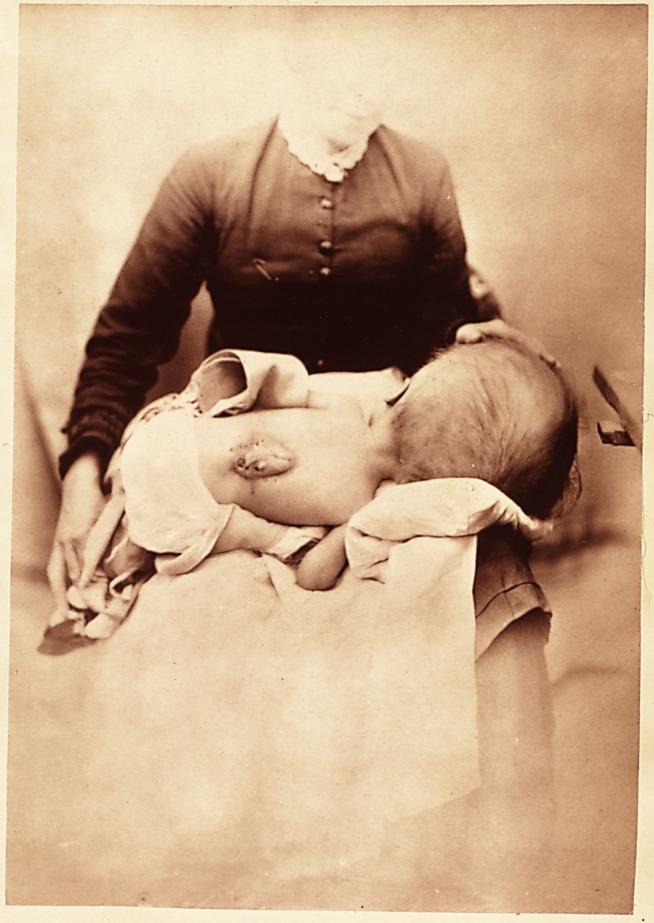# Case of Rupture of the Right Auricle of the Heart

**Published:** 1884-03

**Authors:** George Thompson

**Affiliations:** Medical Superintendent of the Bristol City and County Lunatic Asylum


### CASE OF RUPTURE OF THE RIGHT AURICLE OF THE HEART.

By George Thompson, M.D., L.R.C.P. (Lond.); Medical Superintendent of the Bristol City and County Lunatic Asylum.

Ann Darvill, aged 69; admitted in 1878; died on the 13th of November, 1883.

When admitted, and during the whole time of her residence in the Asylum, she was the subject of very abject melancholy, and took sparingly of food. She was quiet in all her movements.

On the evening of the 12th of November, about six o'clock, shortly after she had taken her tea, I saw her. She was in a state of utter collapse, and had previously complained of pain starting at her heart and shooting down her legs. A stimulant mixture was given her and she was put to bed. Later in the evening she had rallied slightly, but it was quite evident that she was moribund. The pulse throughout the whole period was small and irregular, and no definite alteration of the sounds of the heart, or in the area of cardiac dulness, was up to midnight perceptible. She died at four o'clock on the following morning.

At the autopsy, all the organs were in a condition consistent with health, except the heart. The sac of the pericardium was distended with about four ounces of fluid, partly serum and partly blood. I passed my finger over the surface and discovered a rent in the right auricle sufficient to admit the finger to explore the cavity of the ventricle. On removal of the heart about three ounces of coagulated venous blood was found infiltrated through the areolar tissue surrounding the points of origin of the great vessels. There was no sign of disease in the ruptured parts, but the walls of the right auricle were particularly thin.

NOTE.—It would appear from the length of time—ten hours—which elapsed from the onset of the attack to death, that the rupture occurred at first outside the serous layer of the pericardium, among the roots of the great vessels, and that, by degrees, the opening was enlarged until it extended low enough to rupture the pericardium and fill its cavity, which was the immediate cause of death.

Rupture of the heart is a very rare accident, and is said to affect the ventricles more frequently than the auricles, and the left ventricle more often than the right. In fact, after carefully searching through such books on the subject as are within my reach, I am induced to think this case to be quite unique. Moreover, in most cases recorded, distinct fault of structure, such as aneurism, is invariably reported; but my case is all the more singular, inasmuch as, with the exception of thinness, due probably, to old age, no disease whatever was discovered. The very quiet life which, for five years before, had been led by the patient makes the case all the more remarkable, and one which, in my opinion, deserves to be placed on record.

## Figures and Tables

**Figure f1:**